# Upscaling: efficient generation of human lung organoids from induced pluripotent stem cells using a stirring bioreactor

**DOI:** 10.3389/fbioe.2025.1684315

**Published:** 2025-12-01

**Authors:** Bettina Budeus, Chiara Kroepel, Zehra Fatma Sevindik, Luca Fabian Buttler, Diana Klein

**Affiliations:** Medical Faculty, Institute for Cell Biology (Cancer Research), University of Duisburg-Essen, Essen, Germany

**Keywords:** lung, organoids, upscaling, 3D culture, bioreactor, bioengineering, induced pluripotent stem cells

## Abstract

**Introduction:**

Human induced pluripotent stem cells (iPSCs) can be successfully differentiated into complex (three-dimensional) lung spheroids or organoids and have thus proven to be promising in vitro tools that provide a robust system for simulating lung disease and modeling drug response. We previously described a very simple and practical protocol for producing iPSC-derived lung organoids (iPSC-LuOrgs) in ultra-low attachment plates without relying on a gel-like extracellular matrix.

**Methods:**

Here, we produced these organoids in a stirred-tank bioreactor equipped with a unique membrane stirrer and compared this type of up-scaled and more automated cultivation with the manual variant. Detailed morphological and molecular analyses, including single-cell RNA sequencing, of the differently generated LuOrgs were performed.

**Results:**

Just like in the manual variant, using the bioreactor morphologically comparable lung organoids could be obtained that showed a very similar cellular composition. This type of generation can also be considered as animal-component-free production.

**Discussion:**

These freely floating LuOrgs are now available in large numbers for the investigation of, for example, cancer therapy approaches as a new and patient-oriented in vitro platform.

## Introduction

The lungs are responsible for the basic function of breathing, so any damage to the lungs caused by pulmonary diseases can significantly affect the life expectancy and overall quality of life (Joo et al., 2024). The gradual increase in the number of serious lung diseases, as highlighted by the recent global pandemic resulting from the SARS-CoV-2 virus in 2019 (COVID-19), has increased awareness regarding research on respiratory diseases and sparked interest in the development of suitable human lung mimetics to better understand as well as therapeutically address such pathological events in a patient-centric manner (Zhang et al., 2025). Moreover, the general shortage of donor organs with increasingly serious transplant-related complications, and lack of reliable patient-like *in vitro* models for translational research are encouraging researchers to develop biotechnological methods to produce potentially transplantable lung structures (Derman et al., 2023). However, the complex three-dimensional architecture of the human lung, with its numerous hollow interconnected airways, alveolar units, and pulmonary capillaries, presents particular challenges in tissue engineering for lung (re)generation. Herein, human lung organoids (LuOrgs) in general and particularly those generated from induced pluripotent stem cells (iPSCs) were developed into patient-like *in vitro* model systems for lung diseases and can be largely considered useful alternatives to preclinical animal models (Dye et al., 2015; Foley, 2017). We recently established a relatively simple and feasible method to generate free-floating LuOrgs from human iPSCs through embryonic bodies using ultralow attachment plates without the need for an extracellular matrix (ECM) (Budeus et al., 2025). In this work, we adapted this protocol for use in a unique membrane-stirred tank bioreactor to enable large-scale differentiation of predifferentiated embryoid bodies derived from iPSCs into LuOrgs.

## Materials and methods

### Generation and bioreactor cultivation of LuOrgs

Two commercially available human iPSC lines were used in this work. The SCTi003 iPSC line (iPSC #1) was purchased from StemCell Technologies (Vancouver, BC, Canada), and the iPS01 iPSC line (iPSC #2) was obtained from ALSTEM Inc. (Richmond, CA, United States). The human iPSCs were maintained on Vitronectin XF-coated culture dishes in mTeSR1 medium (#15883465, StemCell Technologies) according to manufacturer protocols under standard cell-culture conditions of 37 °C and 5% CO_2_ in a humidified atmosphere. The cells were tested monthly for *mycoplasma* contamination. The LuOrgs were generated as reported previously (Budeus et al., 2024; Budeus et al., 2025). The harvested iPSCs were counted and subsequently seeded onto ultralow attachment plates (#83.3925.400; SARSTEDT, Nümbrecht, Germany) in mTeSR1 medium at 2,500 cells per 100 µL per well. After 4–5 days, the branching lung organoid (BLO) medium was added at 100 µL per well to the generated embryoid bodies (EBs). After a few more days, the medium was completely changed, and the structures were cultured for the indicated durations with complete medium changes on alternate days. For bioreactor cultivation, the predifferentiated EBs (at 5,000 EBs per 2 L or 6.250 cells/mL) were transferred into BLO medium [DMEM F12 containing GlutaMAX supplemented with 1× N-2, 1× B27, 1× penicillin-streptomycin (5,000 U/mL), 0.4% (v/v) of bovine serum albumin (BSA), 0.4 µM of monothioglycerol, 50 μg/mL of ascorbic acid, 10 ng/mL of KGF/FGF7, 10 ng/mL of FGF10, 50 nM of ATRA, and 3 µM of CHIR-99021] and then into the pre-equilibrated culture vessel of the bioreactor (ComfyCell, BioThrust, Aachen, Germany) at the following parameter values: dissolved oxygen (DO) set point of >/= 50% (actual value: 90–95%); oxygen transfer rate (OTR) of 0.6 (0.8) mmol/(L×h); pH range of 7.2-7.4 membrane gassing at a constant flow of 0.5 L/min, air + 5% CO_2_ on demand, and open-loop off-gas circulation; osmolality range of 310–350 mOsm/kg. The medium was changed once a week, with 1 L of the medium being removed each time and replaced with 1 L of 2× concentrated medium.

### Histology and immunofluorescence

Immunohistochemistry analysis and immunofluorescence staining were performed on the formalin-fixed and paraffin-embedded LuOrgs as described previously (Budeus et al., 2024; Ketteler et al., 2020). At the indicated time points, the LuOrgs were treated with 4% paraformaldehyde in phosphate-buffered saline (PBS) for 30 min, before being subjected to paraffin embedding and sectioning (3–5 µm). The sections were stained with the PAS staining kit (Carl Roth, Karlsruhe, Germany) according to manufacturer protocols for histological evaluations.

### Flow cytometry

Flow cytometry measurements were next obtained as described previously (Budeus et al., 2024; Steens et al., 2020). Briefly, single-cell suspensions were generated by resuspending the LuOrgs in TrypLE (Thermo Fisher Scientific; #12604013) containing 100 U/mL of DNase I, followed by 15 min of incubation at 37 °C. The digestion was stopped by adding PBS containing 2%–5% fetal calf serum (FCS), 2 mM of EDTA, and DNAse I, and the cellular solution was passed through a 70-μm cell strainer. For each FACS staining reaction, 1 × 10^5^ cells were incubated with fluorochrome-coupled antibody (antigen-specific or isotype control) in 100 μL of FACS buffer (5% FCS in PBS) for 20 min at 4 °C. The cells were then washed twice and resuspended in 200 μL of FACS buffer before analysis on a CytoFLEX Platform (Beckman Coulter) using the CytExpert Software (Beckman Coulter). The following antibodies were used in the analyses: S100A4 monoclonal antibody (2G11B4), CoraLite Plus 647 (cat# CL647-66489); CD34 monoclonal antibody (4H11), APC, eBioscience (cat# 17-0349-42); CD31 (PECAM-1) monoclonal antibody (390), APC, eBioscience (cat# 17-0311-82); CD324 (E-Cadherin) monoclonal antibody (DECMA-1), Alexa Fluor 488, eBioscience (cat# 53-3249-82); CD326 (EpCAM) monoclonal antibody (G8.8), PE, eBioscience (cat# 12-5791-82) (all from Thermo Fisher); human Nestin APC-conjugated antibody (cat# IC1259A); alpha-smooth-muscle actin antibody APC (1A4/asm-1, cat# NBP2-34522APC) (both from Bio-Techne Ltd.); CoraLite Plus 488-conjugated caveolin-1 monoclonal antibody QN:Q1604572 (cat# CL488-66067); CoraLite Plus 488-conjugated acetylated tubulin (Lys40) monoclonal antibody (cat# CL488-66200), CoraLite Plus 488-conjugated p63 polyclonal antibody (cat# CL488-12143) (all from Proteintech).

### Single-cell RNA sequencing (scRNAseq) analysis

scRNAseq was performed as described previously (Budeus et al., 2024; Budeus et al., 2025); briefly, single-cell suspensions of the LuOrgs were labeled using the BD®Hu single-cell multiplexing kit (BD, Bioscience; #633781) according to the manufacturer’s instructions. Then, the samples were combined (sample 1 BR + sample 2 BR and sample 3 manual + sample 4 manual) and loaded onto one lane on the BD Rhapsody HT Xpress System aiming for 20.000 cells. Next, the BD Rhapsody library was prepared according to the manufacturer’s instructions and sequenced; cwl-runner provided by BD was used to align and preanalyze the data. In total, 12.526 cells (4.470 BR and 8.506 manual) were identified and further analyses were conducted using R packages (Seurat v.4, Enhanced Volcano, scPubr, fgsea, nVennR). 

### RNA isolation, cDNA synthesis, and quantitative real‐time reverse transcription polymerase chain reaction (RT‐PCR) analysis

The total RNA was isolated using the RNeasy Mini Kit, and cDNA synthesis with integrated genomic DNA removal was performed using QuantiTect Reverse Transcription (Qiagen, Hilden, Germany) according to the manufacturer's instructions. Then, real‐time RT‐PCR analysis was carried out as described previously (Steens et al., 2021; Steens et al., 2020) using the following specific deoxyoligonucleotide primers: bACT forward: CAC​CAT​TGG​CAA​TGA​GCG​GTT​C, reverse: AGG​TCT​TTG​CGG​ATG​TCC​ACG​T; NKX2-1 forward: GCC​ATC​TTC​ACC​CGC​TAC​AA, reverse: TTC​TTG​CTG​CTC​CAC​ACT​GC; TP63 forward: CAG​GAA​GAC​AGA​GTG​TGC​TGG​T, reverse: AAT​TGG​ACG​GCG​GTT​CAT​CCC​T; ID2 forward: CAG​CAG​CAT​CCT​GTC​GAG​TG, reverse: TTG​CTT​CAG​CAC​CAG​CAA​AG; CAV1 forward: CCA​AGG​AGA​TCG​ACC​TGG​TCA​A, reverse: GCC​GTC​AAA​ACT​GTG​TGT​CCC​T; Arg2 forward: GAC​TAC​CGG​AAG​CAT​TTG​GG, reverse: TCA​GGA​GTC​GTT​TGC​AGA​GG; FoxJ1 forward: ACT​CGT​ATG​CCA​CGC​TCA​TCT​G, reverse: GAG​ACA​GGT​TGT​GGC​GGA​TTG​A; Postn forward: CAG​CAA​ACC​ACC​TTC​ACG​GAT​C, reverse: TAA​GGA​GGC​GCT​GAA​CCA​TGC; Acta2 forward: GAC​CCA​GAT​TAT​GTT​TGA​GAC​C, reverse: CCG​ATC​CAG​ACA​GAG​TAT​TTG; SCG1A1 forward: GCT​GAA​GAA​GCT​GGT​GGA​CAC​C, reverse: GCG​TGG​ACT​CAA​AGC​ATG​GCA​G.

### Statistical analysis

Unless otherwise indicated (n = biological replicates), the data were obtained from at least three independent experiments. The data were presented as individual symbols and median values with interquartile range (IQR). Data analyses were performed using R software (R Core Team), and the corresponding tests are indicated where necessary. The statistical significance was set as *p* ≤ 0.05.

## Results

**FIGURE 1:**
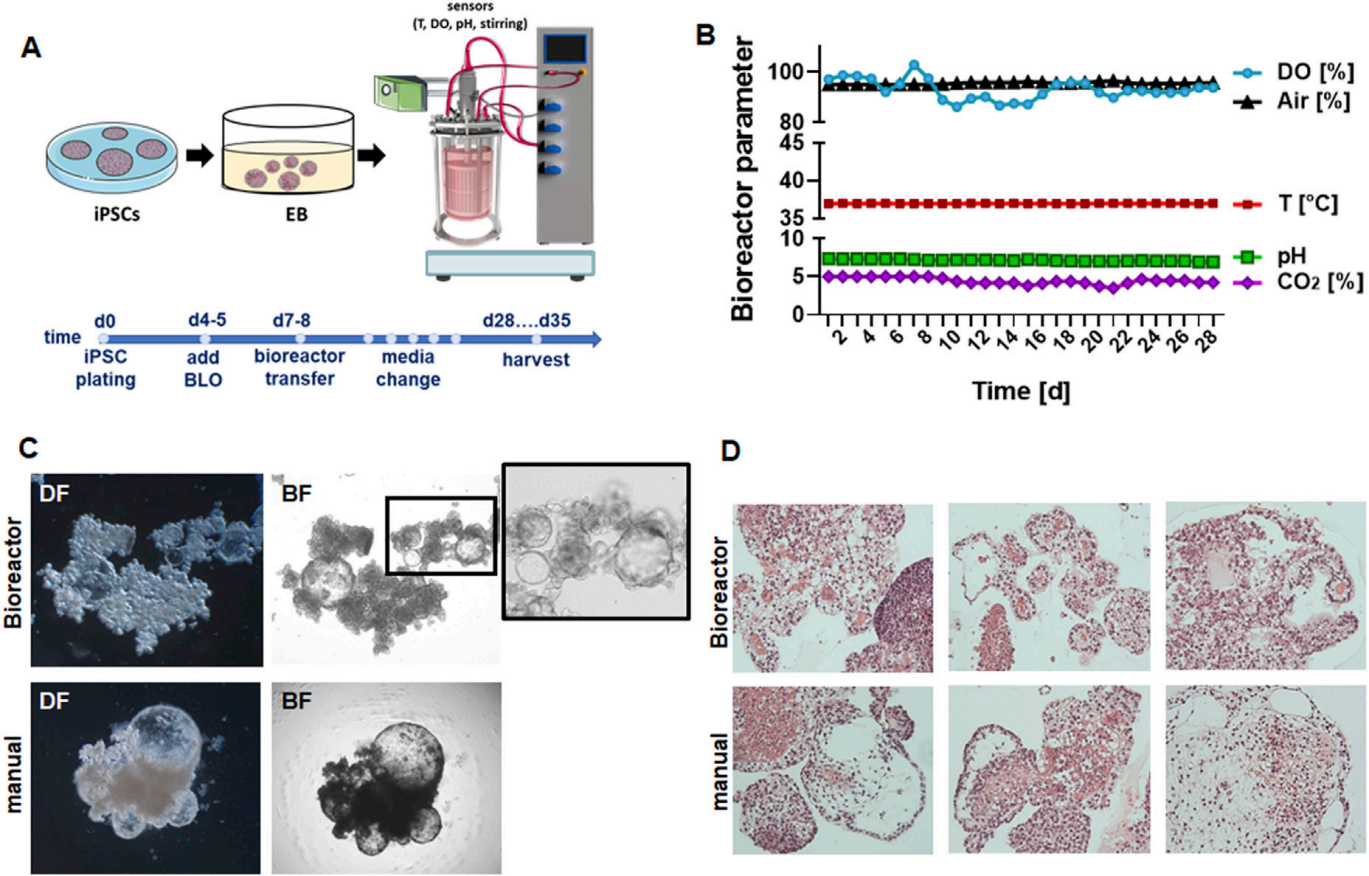
Experimental design and morphological analyses of lung organoids (LuOrgs). **(A)** Scheme for the experimental design including the timeline; induced pluripotent stem cells (iPSCs) were cultured as embryoid bodies (EBs) for 4–5 days in ultralow attachment plates before adding the branching lung organoid (BLO) medium for differentiation (Budeus et al., 2024; Budeus et al., 2025). On day 6 or 7, the formed structures were transferred to the bioreactor (5,000 EBs per 2 L at 6.250 cells/mL) in BLO medium or further cultivated in ultralow attachment plates (manual). **(B)** The indicated bioreactor parameters were recorded continuously (DO, dissolved oxygen). **(C)** Representative dark-field (DF) and bright-field (BF) images of the matrix-free LuOrgs generated from human iPSCs in the bioreactor or manually at the 35-d time point (end of experiment) obtained using the ×10 objective of an inverted microscope. **(D)** Representative periodic acid Schiff histology staining (different lung-like regions) of paraffin-embedded sections of the generated LuOrgs are shown at 10× magnification (examples shown correspond to one of three independent bioreactor runs).

**FIGURE 2:**
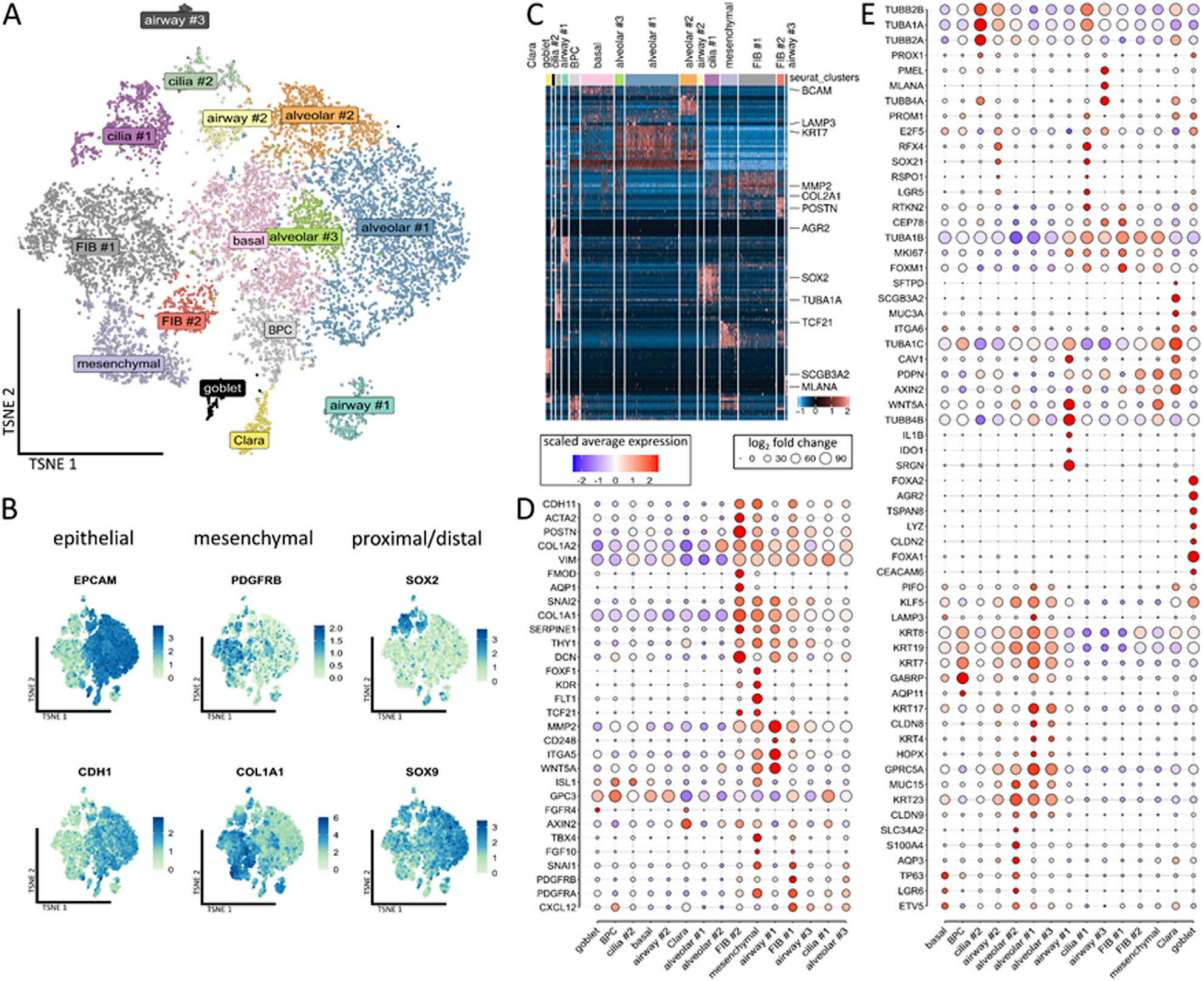
Single-cell analyses of the compositions of the LuOrgs. **(A)** Uniform manifold approximation and projection for dimension reduction (UMAP) of all single cells colored by the identified clusters for N = 4 biological replicates. The cell-type labels for each cluster are based on expression of the canonical cell-type markers displayed in the following plots. **(B)** Expression map of representative epithelial (*EPCAM* and *CDH1*) and mesenchymal (*PDGFRB* and *COL1A1*) marker genes or *SOX2* and *SOX9* for proximal-distal patterning in all clusters. **(C)** Row-normalized heatmap of the top-ten differentially expressed genes per cluster. The important genes are indicated by names. **(D)** Dot plot of the indicated mesenchymal marker genes. **(E)** Dot plot of the epithelial marker genes. The sizes of the dots indicate the percentage of cells in which the corresponding gene was found, while the colors indicate the normalized expression values.

Following our recently published protocols, human iPSCs were plated in ultralow attachment plates (96-well format) for EB generation and subsequent branching lung organoid (BLOB) media treatment (Figure 1A). The predifferentiated structures were transferred to the bioreactor equipped with an integrated membrane stirrer (constant agitation with 80 rpm) to enable unsurpassed gas transfer efficiency via bubble-free aeration while inhibiting foam formation. The DO, pH, temperature, and CO_2_ values were recorded over time for a total of 28 d (Figure 1B), and media changes were performed once a week on days 7, 14, and 21. The generated LuOrgs were harvested after 28 d of bioreactor cultivation (35 d in total) along with the manually cultured LuOrgs in parallel on ultralow attachment plates. Morphological analyses were conducted using phase-contrast microscopy (Figure 1C), immunohistochemistry (Figure 1D), and immunofluorescence (Supplementary Figure S1), which confirmed the efficient generation of airway and alveolar structures representing the desired target tissues. Compared to the manually generated LuOrgs in which lung budding and branching became prominent at approximately 12–14 d post plating (Supplementary Videos S1,S2), the LuOrgs differentiated within the bioreactor appeared to be larger with somewhat fewer alveolar spheres (Figure 1C; Supplementary Video S3). The scRNAseq of the LuOrgs (day 35) generated from the two different iPSC lines finally showed proof of presence of different epithelial and mesodermal lung cell subsets characterizing their respective phenotypes based on bioreactor versus manual cultivation (Figure 2).

Clustering of the single cells revealed the presence of 15 different main cellular subsets (Figure 2A), including epithelial and mesodermal lung cells, as highlighted by the distribution of epithelial *EpCAM*/*CD326*, *ECAD*/*CDH1*, mesodermal *PDGFRB*, and *COL1A1* expressions as well as proximal-distal (*SOX2*-*SOX9*) patterning (Figure 2B). Among the different subsets, the basal cells expressed *TP63*, *LGR6*, and *BCAM* (Figures 2C–E; Supplementary Figure S1). A cluster of designated bronchiolar progenitor cells (BPCs) was located nearby. The simultaneous expressions the alveolar marker *GABRP* and *AQP11* with *KRT7* within this cluster could indicate the bronchioalveolar stem cell nature of these cells differentiating toward bronchiolar cells. The secretory cells and particular goblet cells were identified by the expressions of *FOXA1/A2*, *LYZ*, *ARG2*, *TSPAN8*, and *CEACAM6* located next to the Clara (club) cells expressing *SCGB3A2*, *MUC3A*, and *SFTPD* as well as a cluster of potentially immune conferring cells (designated as airway #1) expressing *IDO1*, *IL1B*, *C3*, and *CXCL8* (Figure 2D; Supplementary Figure S2). Two *TUBA1A*-, *TUBB2B*-, and *RSPO1*-expressing ciliated cell clusters (#1, #2) were identified, where cluster #1 (*RFX4*, *CEP78*, and *MK67*) comprised (differentiating) luminal epithelial cells undergoing ciliogenesis and cluster #2 comprised more mature ciliated cells. In the airway #2 cluster nearby, we observed *KRT23* expression with *RFX4*, *RSPO1*, *TUBA1A*, and *TUBB2B*, indicating suprabasal or “lung umbrella” cells that could play a pivotal role in the development of basal progenitor cells to differentiated luminal cells, thus indicating differentiation toward *SOX2*-positive ciliated cells. Three alveolar and three mesenchymal clusters were further identified, where the alveolar cluster #1 consists of alveolar epithelial cells type 2 (AECII), including AECII-differentiating into AECI cells based on the expressions of *GABRP*, *KRT7*, *MUC15*, and *LAMP3* along with *GPRC5A* and *HOPX* (with the latter two indicating AECI trans-differentiation). Next to the basal cell cluster, the alveolar cluster #2 showed *KRT23*-positive AECI/II lineage-prone cells or specifically AECI/II-differentiating bronchioalveolar stem cells (BASCs) expressing *TP63*, *LGR6*, and *KLF5* along with *ETV5* and *GPRCA5* (as AEC1 lineage markers) as well as *SLC34A2*, *MUC15*, *AQP3*, and *CLDN9*. AECI was also found in alveolar cluster #3 expressing *GPRC5A* and *KRT17* along with *CLDN6* and *CLDN9* as additional alveolar cell markers. Among the mesenchymal cell types, one of the mesenchymal clusters was characterized by general mesenchymal-like gene expressions of *VIM*, *COL1A1/2*, and *CDH11*. Together with the expressions of *PDGFR*-alpha, *TBX4*, *SNAI1*, and *FOXF1*, these cells could represent mesenchymal stem-cell-like cells or more crude fibroblastic precursors including *FLT1*, *KDR*, *TCF21*, and *WNT4A*-positive alveolar fibroblasts. The FIB #1 cluster comprised *PDGFRB*-positive adventitial fibroblasts coexpressing *TWIST1* and *CXCL12*, which in turn could indicate a more activated fibroblast phenotype. The fibroblasts in the FIB #2 cluster were characterized by higher expression levels as well as more reactive ECM-remodeling-related genes like *DCN*, *ACTA2*, *POSTN*, *SERPINE1*, and *HAND1*; these cells were designated as intestinal fibroblasts including myofibroblasts.

**FIGURE 3:**
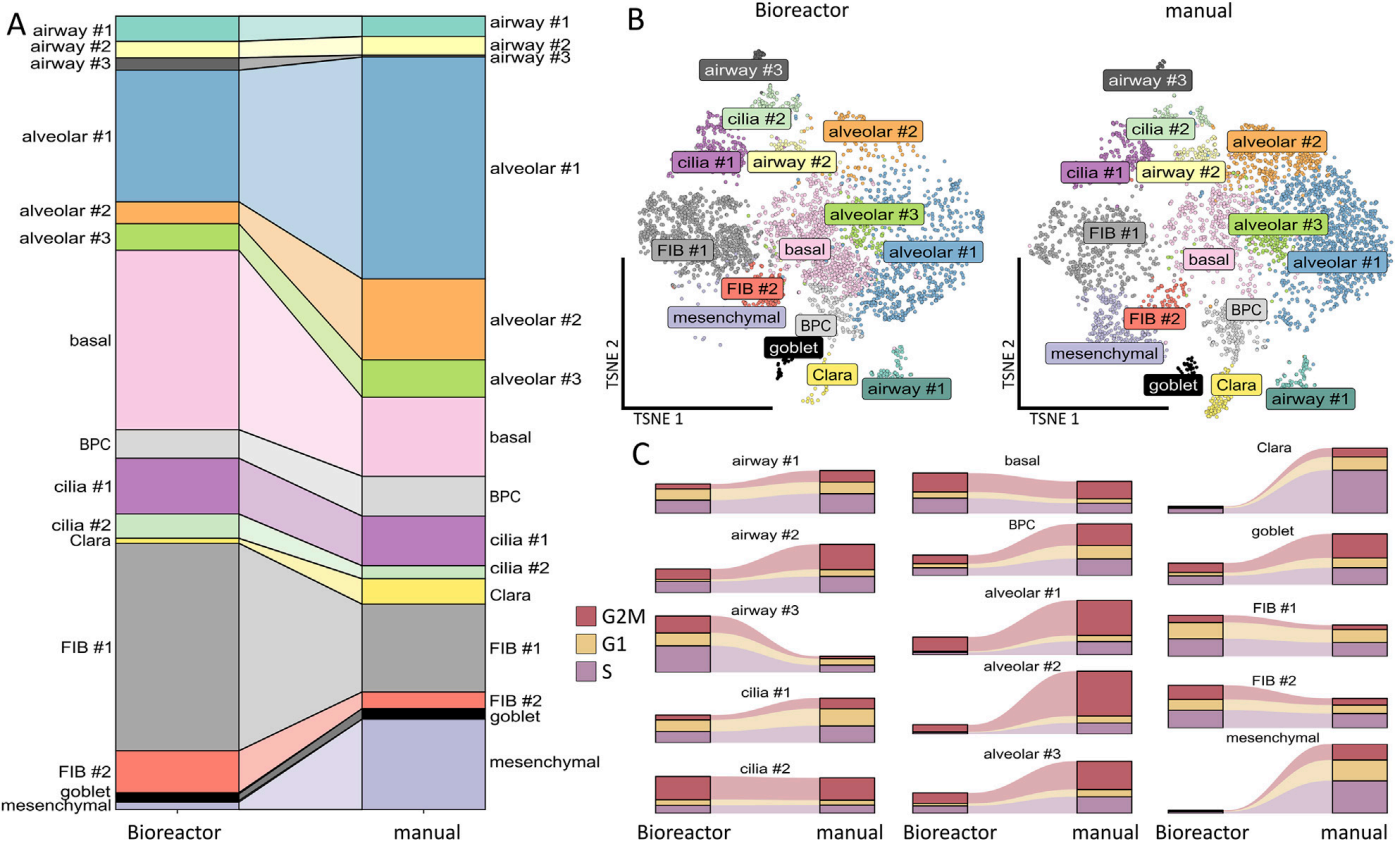
Comparison of bioreactor versus manual differentiation. **(A)** Cluster sizes of the bioreactor-generated versus manually generated LuOrgs. **(B)** UMAP of all single cells colored by the identified clusters per variant. **(C)** Cell-cycle classification per cluster in the bioreactor-generated and manually grown LuOrgs.

**FIGURE 4:**
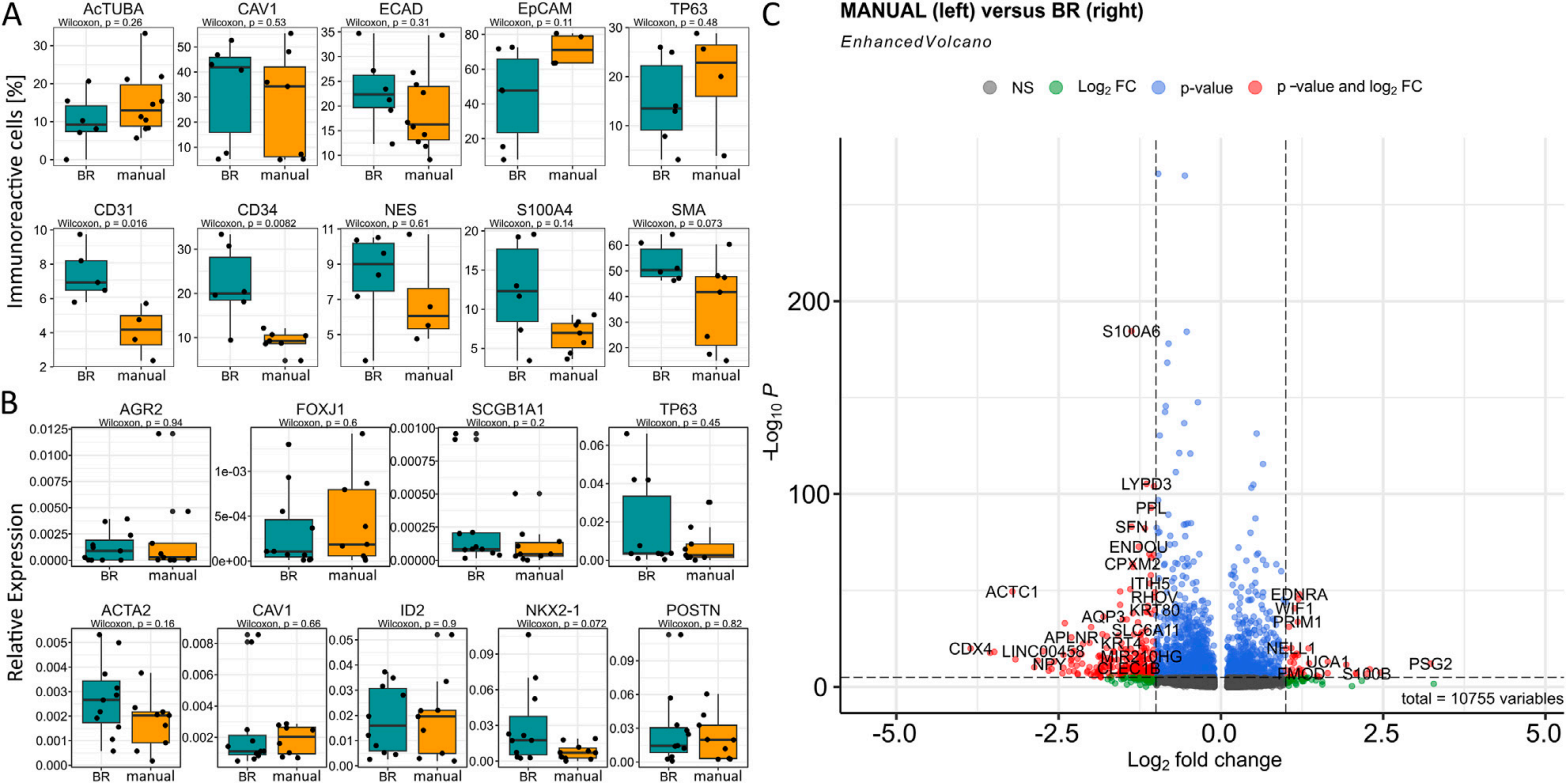
Cell-type-specific marker analysis in the LuOrgs for bioreactor versus manual differentiation. **(A)** Flow cytometry quantification of the indicated marker proteins for various lung cell types for single-cell dissociated LuOrgs. The biological replicates and Wilcoxon signed-rank test values are as indicated. **(B)** Transcript levels as quantified using real-time reverse transcription polymerase chain reaction are shown as relative expression to beta-actin (set as 1). The Wilcoxon signed-rank test values are as indicated. **(C)** Volcano plot of all differentially expressed genes (cluster-independent) obtained from the scRNAseq datasets (n = 4, Mann–Whitney U test). Labels are shown for genes with absolute log2(fold-change) values >1 and *p*-values <0.00001.

With regard to the cellular portions within the clusters according to the cultivation variants, namely, bioreactor versus manual, we observed that the bioreactor-generated LuOrgs contained more basal cells, FIB #1 fibroblasts, and slightly more FIB #2 fibroblasts as well as airway #3 cells (Figures 3A,B). The manually generated LuOrgs contained more alveolar cells (#1, #2, and to a lesser extent #3), more mesenchymal cells, and slightly more Clara cells. The portions of airway #1, #2, bronchiolar progenitor, ciliated (cilia #1, #2), and goblet cells remained similar. The cell-cycle distributions of the respective cells within the clusters were not obviously altered between the two variants but reflected the different portions (Figure 3C). Most cells of all the alveolar, basal, BPC, cilia #2, airway #2, goblet, and mesenchymal clusters were found to be in the G2/M and S phases, indicative of a more proliferative stage, where the cells of the cilia #1, airways #1 and #3, Clara, and FIB #1 and #2 clusters showed greater portions in the G1/G0 phases. We then performed flow cytometry and qRT-PCR analysis using classical lung markers to confirm and refine the potential cellular differences suggested by scRNAseq (Figure 4). Notably, we did not detect any significant differences between the two cultivation variants (Figures 4A,B). From the flow cytometry analyses, we noted a trend toward higher numbers of cells expressing the general epithelial marker EpCAM prominently within the manually cultured LuOrgs, which could reflect the scRNAseq finding of slightly more epithelial cells than mesenchymal cells in these LuOrgs. In the LuOrgs obtained via bioreactor cultivation, we observed a more obvious trend of more mesenchymal (NES, S100A4, and SMA/ACTA2) cells. To further specify the potential differences, the scRNAseq data were analyzed in a cluster-independent manner (Figure 4C); this showed that 1,408 genes were significantly differentially expressed at adjusted *p*-value (p.adj) < 0.001 and fold change (FC) > 1, with 25 genes being upregulated (only) in the LuOrgs of the bioreactor cultures and 1,383 genes being upregulated in the manual variant. The top-10 differentially expressed genes in the bioreactor variant were *PSG2*, *UCNR1*, *PCGEM1*, *S100B*, *SMR3B*, *SOX10*, *EDDM3B*, *UCA1*, *PSG5*, and *AGTR2* while those in the manual culture were *CDX4*, *NANOGNB*, *MAGEA4*, *ACTC1*, *TRPA1*, *ALPG*, *CRHBP*, *LINC00458*, *CRYBA4*, and *NPY*. Thus, the automated differentiation in the stirred-tank bioreactor resulted in numerous LuOrgs of equally good quality as those produced manually.

## Discussion

Among the different lung bioengineering approaches available, those offering scalable expansion and proliferation of iPSCs followed by differentiation into various lung cells with the aim of producing functional reproducible lung tissue constructs using monitoring bioreactors are of central interest (Mahfouzi et al., 2021). Herein, we used a classical stirring bioreactor equipped with the special BioThrust membrane stirrer (Bongartz et al., 2023) (WIPO (PCT) patent application no. W0 2021/152128; International Office patent application no. PCT/EP 2021/052162); the dense hollow-fiber membranes of the stirring module enabled bubble-free and low-shear aeration with gentle mixing at 80 rpm for optimal cell growth and media dispersion. This allowed sufficient differentiation of the inoculated and predifferentiated iPSC-derived EBs into LuOrgs in large numbers.

As a more ethical alternative to animal models, (spheroidal) LuOrgs as miniature 3D lung structures offer a higher degree of tissue complexity and heterogeneity, representing a faster and more viable method for accurately simulating and ultimately modulating human lung physiology (Joo et al., 2024; Zhang et al., 2025; Vazquez-Armendariz and Tata, 2023). The classical so-called airway organoids comprise basal cells, functional multiciliated cells, mucus‐producing secretory cells (particularly goblet cells), and club cells (Sachs et al., 2019). These organoids are generated from normal lung tissues following bronchoalveolar resection or lavage material (Sachs et al., 2019) or even from normal tissues adjacent to lung cancers (Zhang et al., 2025) via enzymatic digestion of the minced tissues; the resulting tissue suspensions are plated in growth-factor-reduced membrane extracts prior to cultivation in special media formulations (Sachs et al., 2019; Kim et al., 2019). The organoids derived from lung cancer tissues can recapitulate the tissue architecture while maintaining the genomic alterations of the original tumors (Kim et al., 2019; Strocchi et al., 2025). These patient-derived organoids are excellent models for research and are excellent predictive tools in clinical practice, e.g., assessment of patient-specific drug responses (Sachs et al., 2019; Kim et al., 2019; Strocchi et al., 2025). Airway-like human LuOrgs can also be obtained from human iPSCs following induction of definitive endoderm and anterior foregut endoderm as well as 3D embedding of self-aggregating foregut spheroids in Matrigel (Miller et al., 2019); bronchial-like structures were then obtained following cultivation with a low concentration of fetal bovine serum in the presence of FGF10 to yield human LuOrgs that formed airway-like structures and cell types surrounded by mesenchymal populations. When the embedded foregut spheroids were cultured for an extended duration in the presence of FGF7, CHIR-99021, and ATRA, so-called bud-tip progenitor organoids were obtained that formed patterned branch-like structures resembling proximal airway and distal bud-tip regions (Miller et al., 2019). We previously developed an easy-to-use and highly reproducible modification of this four-step protocol for ECM-free generation of LuOrgs from human iPSCs (Budeus et al., 2024; Budeus et al., 2025). The quality and robustness of these LuOrgs as potential *in vitro* platforms for lung diseases, particularly radiation-induced lung injury, were described for manually generated structures (Budeus et al., 2024). The starting point in this method is the spontaneous generation of EBs from iPSCs in an ultralow attachment suspension system that differentiate into proximal and distal branched airway epithelial structures after treatment with BLO medium. In the present work, we automated and scaled up this process by initially transferring the partially differentiated EBs into the bioreactor. Unlike similar stirred-tank bioreactors, our system is equipped with a special membrane module for gassing. Further efforts by our research group focus on complete differentiation in the bioreactor, i.e., proliferation of iPSCs and subsequent initiation of differentiation directly in the bioreactor culture vessel, which would further simplify and accelerate the process.

Upscaling along with automated generation and maturation of high-fidelity organoids using bioreactors is becoming increasingly important in lung bioengineering (Derman et al., 2023; Wilkinson et al., 2017; Licata et al., 2023). The use of stirred bioreactors for these cultures generally involves developing and optimizing environmental conditions that can provide optimal cues for growth and 3D maturation of the desired (lung) tissue structures, such as oxygenation, mechanical and fluidic activation, and nutrition gradients (Derman et al., 2023). These bioreactors are typically equipped with a motor-driven stirrer positioned within a cylindrical culture vessel with sampling ports and a heating unit. The O_2_ and CO_2_ concentration adjustments as well as pH regulation are carried out automatically via sensor probes coupled to air and CO_2_ supplies. Most eukaryotic cell cultures are performed with DO levels of approximately 20%–50%. We performed our first experiments with DO of ∼90% that was recommended by the manufacturer; the CO_2_ level was set at 5%, pH was regulated to 7.4, and controlled factors remained constant over the cultivation period of 28 d. However, in the future, it is important to determine how the non-compounded oxygen levels in the media impact differentiation, particularly the oxygen-sensitive structures like those present in the lungs (Klein, 2025; van Vliet et al., 2021; Zacharias, 2021).

Given the particular structural complexity of organoid tissue multilayers, the oxygen and nutrition availability improvements accomplished by stirred bioreactors limit the development of hypoxic and/or necrotic cores within the formed multicellular structures (McMurtrey, 2016). However, oxygenation (for example) was found to be the driving factor for epithelial differentiation in air–liquid interface cultures, which is a rather expensive, laborious, and challenging task when scaling for increased throughput applications (Kouthouridis et al., 2021; Guénette et al., 2022). At the differentiation-promoting air–liquid interface, cells usually experience “normoxic” oxygen levels, which is defined as the concentration of oxygen-saturated fluid at ambient O_2_ (0.21 mol/m^3^) (Kouthouridis et al., 2021). The oxygen concentration and pressure are generally pivotal elements of the microenvironment for lung development, so control and variation of these parameters clearly influence the production of multicellular organoids with the desired architectural complexity (Ozan et al., 2025). Accordingly, the differentiation of iPSCs into LuOrgs was perfomed in an oxygen- and pressure controlled microenvironment using low (5%) oxygen levels until the generation of early LuOrgs that mimics the lower fetal lung pressure (approximately 20–30 mmHg) and increasing (10%) oxygen levels until further LuOrg maturation, similar to the increasing oxygen concentrations and lung pressures at birth (approximately 50–60 mmHg) (Ozan et al., 2025). Gene and protein expression levels along with pathway analyses revealed upregulation of the lung-development-specific pathways of the LuOrgs compared to structures growing under normal culture conditions, strongly highlighting the impact of oxygen level on lung development.

Tissue oxygenation within the human body is a tightly regulated process (Place et al., 2017). The physiological partial pressure of arterial oxygen is maintained at approximately 80–100 mmHg (10.8%–13.5% O_2_), which equates to 0.13 mmol of unbound oxygen per liter of blood (at sea level). The physiological oxygen tension in the lungs (alveolus) is approximately 104–108 mmHg (Zacharias, 2021; Zhang et al., 2016; Rogers et al., 2025). Within the tissues, this value decreases to approximately 40 mmHg owing to cellular oxygen consumption, which in turn causes an oxygen gradient and pulls the DO by a short distance from the capillaries to the oxygen-consuming cells (Place et al., 2017). In cell cultures, regulatory mechanisms concerning “oxygen delivery” are missing; under standard culture conditions of 37 °C, 5% CO_2_, and 100% humidity (water vapor supplied by a water bath in the incubator), the final proportion of oxygen in the incubator is 18.6% and not 20.95% (volume/volume fraction of oxygen in dry air with 78.09% nitrogen, 0.93% argon, and 0.039% carbon dioxide) (Place et al., 2017). This 18.6% oxygen proportion is equivalent to an oxygen tension or oxygen partial pressure (pO_2_) of 138 mmHg and is designated as the experimental normoxic value (Martinez et al., 2019). However, various types of cells cultured in standard medium volumes are supposed to be hypoxic owing to oxygen consumption exceeding the diffusion rate, although cell culture is generally considered a microenvironment with high oxygen (Tan et al., 2024). The supply and demand relationships of oxygen in cell cultures have been described extensively. Under standard culture conditions, for example, when 1 mL of the medium is used per well (12-well plate), the depth of the medium is estimated as 2.4–2.9 mm. For a cell diameter range between 40 and 100 μm, this means that the oxygen must diffuse over a distance equivalent to between 27 and 58 cell diameters to reach the cells, meaning that the diffusion distances are higher compared to the *in vivo* situation (Tan et al., 2024). Therefore, standard cell-culture conditions are considered to be a significant barrier to oxygen supply, which is clearly a negligible limitation when using stirred bioreactors. The main advantages of these systems are the improved mass transfer and enhanced oxygenation by media agitation, which can finally support increased cell survival and accelerated differentiation (Licata et al., 2023; Avena et al., 2025; Liu et al., 2025).

Thus, the DO concentration should be considered one of the most critical parameters when designing bioreactors, particularly with respect to the large-scale or high-density cultivation of lung cells as intended herein (Wang et al., 1994). In the meantime, the DO levels investigated here are only limited to the range approximately 100% air saturation (6.8 mg/L), so the far-reaching effects of DO levels on different cell types have not been investigated in detail. Given the fact that different tissues have unique ranges of physiological normoxia depending on their specific oxygen demands or even the needs of individual lung cell types at least during organoid development, using a specific oxygen concentration may not be representative of the *in vivo* environment and needs further evaluation in future experiments.

Excessive hydrodynamic forces could further negatively impact delicate structures such as the cilia (Mahfouzi et al., 2021; Licata et al., 2023; Ovando-Roche et al., 2018). Lung organoids may be considered as “very” shear-sensitive organoids as the resulting alveolar vesicles may collapse more readily owing to movements in the reactor. Although the cellular composition does not indicate a significant difference in the numbers of alveolar epithelial cells, morphological examination of the obtained (and not yet fixed) LuOrgs suggest that fewer alveoli in the form of small blisters were present in the bioreactor cultivation variant. However, these effects could be minimized by establishing optimal and/or minimal stirring conditions. Perhaps these structures could be stabilized by using a more semisolid medium in the bioreactor, which is not necessary with the ultralow attachment plates given the lack of turbulence during parallel cultivation. Furthermore, co-cultivation with endothelial cells, for example, through simultaneous differentiation and/or intercalation with endothelial cells during the differentiation process, could promote stabilization of these structures while ensuring that the LuOrgs are closer to the *in vivo* situation through the formation of endothelial–alveolar barriers.

The lack of endothelial cells in addition to the lack of mesenchymal and immune cells represents a general limitation of (lung) organoids to fully recapitulate the cellular composition of the desired target tissue. This could be improved and the complexity could be increased through intercalation or simultaneous co-cultivation of endothelial cells or fibroblasts to foster generation of certain structures, e.g., the alveolar compartment (Ament et al., 2025; Qadir et al., 2025; Goltsis et al., 2024; Tamai et al., 2022; Yamamoto et al., 2017). Through the EB-induced differentiation used herein, various lung epithelial and mesenchymal cells that provide structural and instrumental support, particularly to the alveolar epithelium, may be differentiated simultaneously; these are arranged nicely in a lung-typical structure and were cultured without the need of an ECM. A recently published study has described the generation of vascularized LuOrgs with efficient differentiation and specification of both the endodermal derivatives and organotypic endothelial and mesenchymal populations in an elegant manner (Miao et al., 2025); the starting point in this work was also reported as EBs. The resulting LuOrgs were sufficiently vascularized (i.e., they contained organotypic endothelium and mesenchyme) and exhibited improved cell type diversity, 3D architecture, cell survival, and maturation (Miao et al., 2025). As alveolar cells are closely related to the microvasculature, forming an efficient air–blood structure in which the capillary endothelium is in direct contact with the alveolar epithelium through the connective tissue layer would resemble higher levels of cell differentiation, maturation, and function than those seen in actual lungs.

## Data Availability

The datasets presented in this study can be found in online repositories. The names of the repositories and accession numbers can be found in the article/Supplementary Material.
