# Supplementary material for: Upscaling: efficient generation of human lung organoids from induced pluripotent stem cells using a stirring bioreactor
Source: Front Bioeng Biotechnol. 2025 Dec 1;13:1684315. doi: 10.3389/fbioe.2025.1684315 (PMC12702919; doi:10.3389/fbioe.2025.1684315)
Supplement: Supplementary file 2 [file DataSheet1.pdf]

Supplementary Material

Upscaling: Efficient generation of human lung organoids from induced pluripotent stem cells using a stirring bioreactor

Bettina Budeus[1], Chiara Kroepel[1], Zehra Fatma Sevindik[1], Luca Buttler[1], Diana Klein1,*

[1]Institute for Cell Biology (Cancer Research), Medical Faculty, University of Duisburg-Essen, Essen, Germany

* Correspondence:

Prof. Dr. rer. nat. Diana Klein; Diana.Klein@uk-essen.de

(https://orcid.org/0000-0002-1770-443X)

Data availability

The single cell RNA-seq data have been deposited at Gene Expression Omnibus (GEO) and are publicly available as of the date of publication (accession number: GSE301233). Any additional information required to reanalyze the data reported in this paper is available from the corresponding author upon request.

Supplemental Movies

Supplemental Movie 1 and 2:

Continuous live cell imaging of plated iPSCs to visualize lung budding and branching (Movie 1: iPSC#1; Movie 2: iPSC#2) was performed starting 10 days after initial cell plating and subsequent differentiation media treatment for additional 96 hours (manual culture version).

Supplemental Movie 3:

Video recording of the formed lung organoids in the bioreactor shortly before the end of cultivation (day 28). The membrane stirrer can be seen in the upper part of the rounded culture vessel. The vessel is illuminated from below with a cell phone flashlight.

Supplemental Figure S1

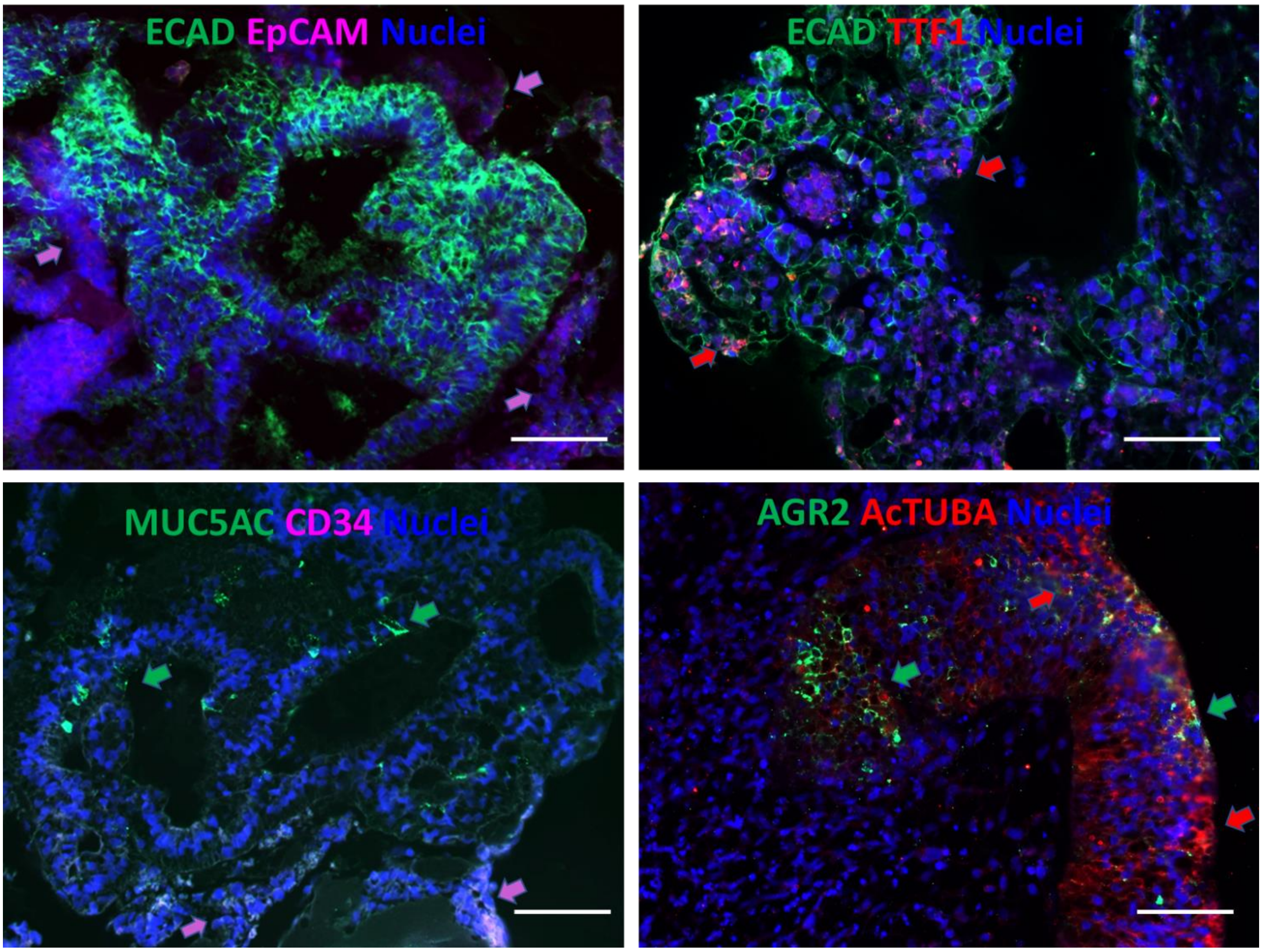

Immunofluorescent analysis of free-floating LuOrgs generated matrix-free from human iPSCs in the Bioreactor at the 35-day time point (end of experiment). Representative immunofluorescent images of indicated lung cell type-marker proteins using the 20x objective of an inverted microscope are exemplarily shown. Scale bar: 150µm.

Supplemental Figure S2

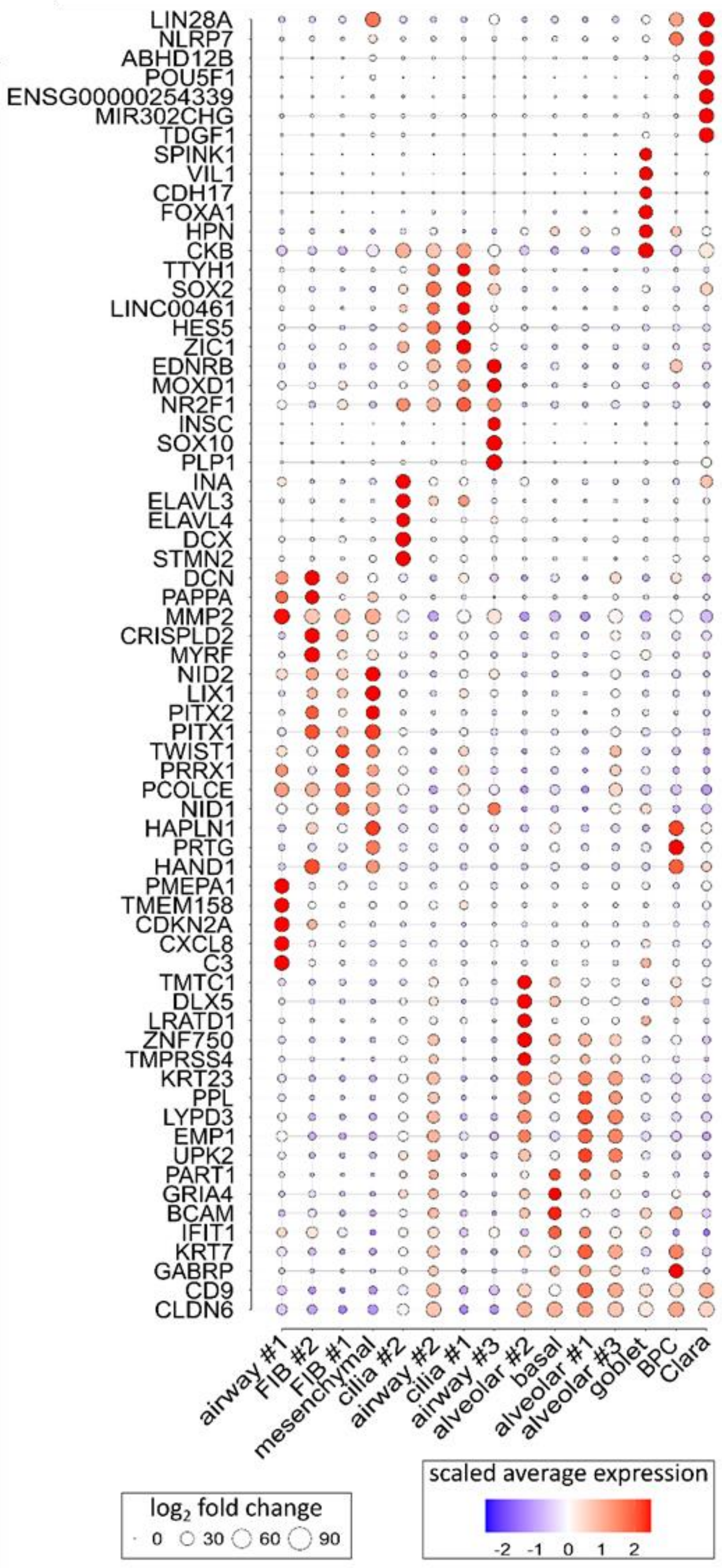

Molecular differentiation of generated lung cells. Dot plot of the top five differentially expressed genes per cluster. Size of the dots indicate the percentage of cells in which this gene was found, the color indicates the normalized value of the expression.
